# Correlation between Diameter of Yolk Sac on Transvaginal Ultrasonography at 6-12 Weeks of Gestation and Adverse Pregnancy Outcomes

**DOI:** 10.5935/1518-0557.20230052

**Published:** 2024

**Authors:** Haitham Fathy Gad, Mohamed Mahmoud Salman, Magdy Hassan Koleib, Aya Ibrahim Nada, Mohamed Abdellatif Daoud

**Affiliations:** 1 Lecturer of Obstetrics and Gynecology, Ain Shams University, Cairo, Egypt; 2 Professor of Obstetrics and Gynecology, Ain Shams University, Cairo, Egypt; 3 Resident of Obstetrics and Gynecology, Elgalaa Military Hospital, Cairo, Egypt

**Keywords:** Yolk sac Diameter, Adverse Pregnancy Outcome, early pregnancy loss

## Abstract

**Objective:**

To evaluate the correlation between yolk sac diameter at 6 to 12 weeks of gestation measured via transvaginal ultrasound and adverse pregnancy outcomes.

**Methods:**

This prospective cohort study was conducted at the Ain Shams University Maternity Hospital from July 1, 2019 to January 30, 2020. It included 120 pregnant women attending the outpatient clinic at 6 to 12 weeks of gestation. Transvaginal ultrasound was performed to measure inner yolk sac diameter. Normal diameter was considered to be 2-5 mm. Cases were followed up in routine antenatal care until the 16^th^ week of gestation.

**Results:**

Significant associations were found between maternal age and yolk sac diameter; yolk sac diameter and early miscarriage; a high percentage of cases of positive fetal life occurred when a normal yolk sac diameter (2-5 mm) was present (*p*<0.001); in yolk sac diameters <2mm positive fetal life was 0.0% and negative fetal life was 42.9%; in yolk sac diameters of 2-5mm positive fetal life was 81.1% and the negative fetal life was 7.1%; and in yolk sac diameters >5mm positive fetal life was 18.9% and negative fetal life was 50.0% (*p*<0.001), x^2^ 60.094; and the best cutoff value for yolk sac diameter was >0.56, with a sensitivity of 78.6%, a specificity of 84.3%.

**Conclusions:**

We found a highly significant correlation between yolk sac diameter and early pregnancy loss.

## INTRODUCTION

The yolk sac is the first extra embryonic structure that becomes visible on ultrasound examination within the gestational sac. It acts as the primary route of exchange between the human embryo and the mother before placental circulation is established. It provides nutritional, metabolic, endocrine, immunologic, and hematopoietic support during organogenesis in embryonic life, and is considered to reach its highest level of functional activity between the 4^th^ and 7^th^ week of embryonic development (Pereda & Niimi, 2008).

The yolk sac is a critical landmark that identifies a true gestational sac (Kurtz *et al*., 1992). Sonography shows the yolk sac as a round structure made up of an anechoic center bordered by a regular well-defined echogenic rim. The diameter of the yolk sac is usually 2-5 mm and increases in size up to the 10^th^ week of gestation (Küçük *et al*., 1999).

Transvaginal sonographic diagnosis of a blighted ovum is certain when the mean gestational sac diameter exceeds 8mm without a yolk sac or when the mean gestational sac diameter exceeds 16mm without an embryo. Transabdominally, a gestational sac greater than 20mm without a yolk sac or 25mm without an embryo is diagnostic of a blighted ovum (Levi *et al.,* 1988).

Threatened and spontaneous abortions represent together the most common complications of early pregnancy. Only 1/3 of the total number of conceived embryos continue further development (Hakim *et al*., 1995).

Size of the yolk sac has been correlated with spontaneous abortion, although studies have published conflicting conclusions on the subject. According to some authors, pregnancy outcome is poor when an enlarged or small yolk sac is present. Other authors have concluded that pregnancy can have a normal outcome in spite of the presence of an enlarged or small yolk sac (Geetanjali *et al*., 2016).

## MATERIAL AND METHODS

This prospective cohort study was conducted at the Ain-Shams University Maternity Hospital (ASUMH) (Special Fetal Care Unit) from July 1, 2019 to January 30, 2020. It included 120 healthy pregnant women at 6 to 12 weeks of gestation attending our outpatient clinic for antenatal care. The study aimed to evaluate the correlation between yolk sac diameter at 6 to 12 weeks of gestation measured via transvaginal ultrasound and adverse pregnancy outcomes.

### Eligibility Criteria

Inclusion criteria: pregnant woman aged 18-35 years, with a gestational age of 6-12 weeks, primiparas or multiparas, with a body mass index of 18-30. Exclusion criteria: pregnancy-related complications (DM, HTN), multiple pregnancy, obstetric complications in a previous pregnancy, uterine abnormalities, bleeding in early pregnancy.

### Ethical Considerations

This study was granted approval by the Ethics Committee of the Department of Obstetrics and Gynecology, Faculty of Medicine, Ain Shams University. The study was conducted in accordance with current clinical protocols, relevant policies, and requirements and regulations in effect at the Ain Shams University Maternity Hospital. Informed consent was taken from all participants before enrollment, and they were explained the purpose and procedures of the study. The principal investigator obtained written consent from every study participant prior to the start of the study. All laboratory specimens, evaluation forms, reports, video recordings and other records that left the site did not contain personal information to safeguard the patient’s identities. The study was funded by the principal investigator.

### Study procedure

Full patient history was taken to analyze eligibility, including personal information such as name, age, occupation and special habits; present and past history; family history; drug history; obstetric history including gravidity, parity, and gestational age; menstrual history based on Naegele’s rule, a standardized way of calculating the due date when assuming a gestational age of 280 days at childbirth. The rule estimates the expected date of delivery (EDD) by adding a year, subtracting three months, and adding seven days to the woman’s last menstrual period. The result is approximately 280 days (40 weeks) from the start of the last menstrual period. Another method is by adding 9 months and 7 days to the first day of the last menstrual period.

General examination: assessment of vital data such as temperature, blood pressure, heart and lung auscultation to exclude contraindications to anesthesia. Abdominal examination: assessment of abdominal tenderness and previous caesarean section scar, if present. Healthy pregnant women meeting the inclusion and exclusion criteria were referred to the special fetal care unit for transvaginal ultrasound (TVUS) examination.

### TVUS

A senior sonographer operated a Samsung H60, Convex Pro = CV1 = 8MHz, prob 4.9 MHz ultrasound scanner to obtain coronal and sagittal views of the areas of interest. A systematic approach was used for performing TVUS. First the uterus was scanned, then the adnexa, and finally the cul-de-sac. The gestational sac and yolk sac were identified. The inner yolk sac diameter was measured by placing calipers at the inner margin. The yolk sac diameter was measured based on two cross sections in two dimensions. The range of normal diameter was considered to be 2-5 mm. A large yolk sac was defined as a yolk sac with a diameter of more than 5mm, while a small yolk sac had a diameter of less than 2mm.

The patients were followed up in the routine antenatal care program and thorough ultrasound examination including fetal biometry using the Hadlock method was performed at 16 weeks of gestation (fetal heart rate and fetal life, fetal biparietal diameter, abdominal circumference, femur length, head circumference). Measures were taken to confirm normal growth and correct gestational age matching the dates of the last menstrual period (LMP). Fetal weight was estimated, the amniotic fluid index was calculated, and location of the placenta was evaluated.

### Measured outcomes

**Primary outcome:** association between yolk sac diameter and early pregnancy loss

**Secondary outcome:** association between yolk sac diameter and maternal demographic data and fetal biometry

### Statistical analysis

Recorded data were analyzed using the Statistical Package for Social Sciences, version 20.0 (SPSS Inc., Chicago, Illinois, USA). Quantitative data were expressed as means ± standard deviation (SD). Qualitative data were expressed as frequencies and proportions. Independent-samples t-test of significance was used when comparing between two means. The Mann-Whitney U test was applied for two-group comparisons in non-parametric data. The chi-square (x^2^) test of significance was used in order to compare proportions between qualitative parameters. Receiver operating characteristic (ROC) analysis was used to find the overall predictivity of parameters and to find the best cut-off value with detection of sensitivity and specificity at such cut-off value. The confidence interval was set at 95% and the margin of error accepted was set at 5%. Therefore, the *p*-value was considered significant as follows: *p*-value <0.05 was considered significant; *p*-value <0.001 was considered highly significant; *p*-value >0.05 was considered non-significant.

## RESULTS

**Figure 1:**
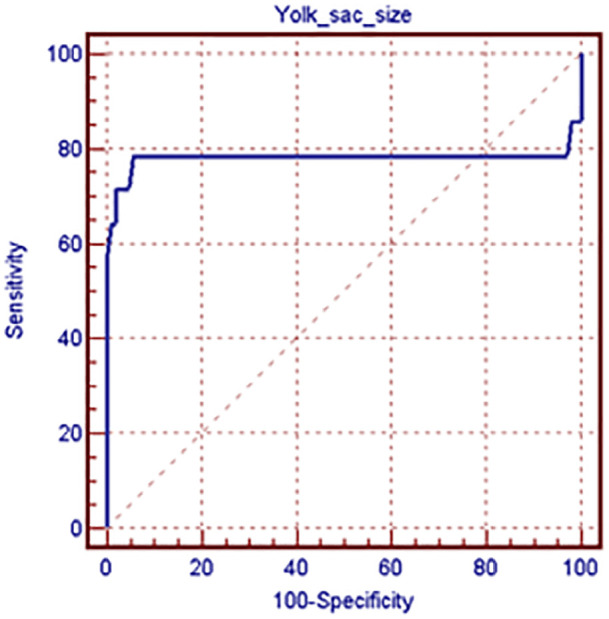
Receiver operating characteristic (ROC) plot for prediction of miscarriage using yolk sac size. The ROC plot was used to define the best cutoff value of yolk sac size, which was >0.56, with a sensitivity of 78.6%, specificity of 84.3%, positive predictive value of 74.7%, negative predictive value of 87.1%, and diagnostic accuracy of 78.2%.

Tables 1, 2 3,4,5,6,7,8 and 9 and figure 1 detail the results.

**Table 1:** Distribution of pregnant women according to their baseline characteristics regarding age, body mass index, parity (n=120).

Baseline characteristics	Total (n=120)
**Age (years)**	18-42 [27.28±5.31]
**Body mass index(kg/m^2^)**	18-30 [25.76±3.00]
**Parity**	0-5 [2 (2)]^#^
**Parity level**	
Multipara	97 (80.8)
Primipara	23 (19.2)

#*Median and Interquartile range (IQR).*

**Table 2:** Distribution of pregnant women according to demographic data of scan in the first visit between 6-12 weeks of gestation(n=120).

1^st^ Trimester Scan	Total (n=120)
Systolic Blood Pressure (mmHg)	100-180 [115.92±11.47]
Diastolic Blood Pressure (mmHg)	50-90 [74.83±8.50]
Gestational age (weeks)	6-12 [8.48±2.03]
Gestational sac (mm)	15.5-93.6 [38.4±15.7]
Crown rump length(mm)	2.1-447 [26.1±42.4]
Yolk sac diameter(mm)	0.9-9.1 [4.10±1.4]

**Table 3:** Yolk sac diameter ranges according to demographic data in the first trimester scan.

Demographic data	Yolk sac <2mm (n=6)	Yolk sac 2-5mm (n=87)	Yolk sac >5mm (n=27)	ANOVA	*p*-value
**Maternal age (years)** Mean±SD Range	34.33±4.46 30-42	26.28±5.06 18-39	28.93±4.81 20-35	9.265	<0.001^**^
Parity Median (IQR) ¥ Range	2 (1) 1-3	2 (2) 0-4	2 (2) 0-5	0.889#	0.414
Body mass index Mean±SD Range	25.13±4.64 19.9-30	25.49±2.89 19-30	26.77±2.85 19-30	2.030	0.136

**Table 4:** Yolk sac diameter ranges according to gestational sac diameter (mm) and crown rump length(mm) in first trimester scan.

	Yolk sac <2mm (n=6)	Yolk sac 2-5mm (n=87)	Yolk sac >5mm (n=27)	ANOVA	*p*-value
**Gestational Sac diameter (mm)** Mean±SD Range	35.42±8.60 26.5-49.7	38.84±15.28 17.8-77	37.44±18.24 15.5-93.6	0.191	0.827
**Crown rump length(mm)** Mean±SD Range	18.83±8.94 7.5-33.1	23.97±17.95 2.7-70.3	19.56±17.24 2.1-58.2	0.810	0.447

*F-One Way Analysis variance; p-value>0.05 NS.*

**Table 5:** Distribution of pregnant women according to fetal life (n=120).

Pregnancy outcome	Total (n=120)
Positive fetal life	106 (88.3)
Negative fetal life	14 (11.7)
Total	120 (100.0)

**Table 6:** Distribution of 120 pregnant women according to second trimester visit (n=120).

2nd Trimester Scan	Total (n=120)
Systolic Blood Pressure (mmHg)	100-180 [115.92±11.47]
Diastolic Blood Pressure (mmHg)	50-90 [74.83±8.50]
Gestational age of second scan (weeks) (n=106)	16-24 [18.56±2.77]
Biparietal diameter (n=106)	29-82 [41.8±10.8]
Abdominal circumference (mm) (n=106)	11.1-201.8 [128.3±37.9]
Femur-length (mm) (n=106)	18-52 [29.5±8.2]
Estimated fetal weight (g) (n=106)	110-820 [308.67±180.64]
Gestational age by US (n=106)	15-24 [18.36±2.78]
Amniotic fluid index (mm) (n=106)	77.1-228 [136.5±36]

**Table 7:** Correlation between yolk sac diameter in first trimester scan and pregnancy outcome in the second trimester scan.

Yolk sac (mm)	Pregnancy outcome				Total		Chi-square test	
	Positive fetal life		Negative fetal life					
	No.	%	No.	%	No.	%	x^2^	*p*-value
Yolk sac <2mm	0	0.0%	6	42.9%	6	5.0%	60.094	<0.001^**^
Yolk sac 2-5mm	86	81.1%	1	7.1%	87	72.5%		
Yolk sac >5mm	20	18.9%	7	50.0%	27	22.5%		
Total	106	100.0%	14	100.0%	120	100.0%		

*Using: Chi-square test; p-value<0.001 HS.*

**Table 8:** Correlation between yolk sac diameter in first trimester scan and biparietal diameter(mm), abdominal circumference (mm) femur length (mm), estimated fetal weight (gm) gestational age by us (weeks) amniotic fluid index(mm) in the second trimester scan.

	Yolk sac 2-5mm (n=87)	Yolk sac >5mm (n=27)	t-test	*p*-value
**Biparietal diameter(mm)** Mean±SD Range	41.35±10.14 29-a82	43.99±13.33 29.1-a82	0.973	0.326
**Abdominal circumference (mm)** Mean±SD Range	126.81±34.90 82-a201.8	139.73±40.62 82-a201.8	2.090	0.151
**Femur length** Mean±SD Range	32.24±30.20 18-a299	31.50±8.94 19.1-a48	0.012	0.913
**Estimated fetal weight (gm)** Mean±SD Range	296.53±172.63 110-a820	360.85±208.52 138-a730	2.078	0.152
**Gestational age on US (weeks)** Mean±SD Range	18.20±2.69 15-a24	19.05±3.12 15-a24	1.531	0.219
**Amniotic fluid index (mm)** Mean±SD Range	136.44±36.12 77.1-a228	137.00±47.28 11-a220	0.003	0.954

*T-Independent Sample t-test; p-value >0.05 S.*

**Table 9:** Correlation between yolk sac diameter (mm) and all parameters using Pearson’s correlation coefficient in the study group.

Parameters	The Yolk sac size	
	R	*p*-value
Age (years)	0.023	0.800
Parity	-0.055	0.549
Body mass index	0.328	0.012^*^
Systolic Blood Pressure (mmHg)	0.111	0.227
Diastolic Blood Pressure (mmHg)	0.061	0.505
Gestational age (weeks)	-0.305	0.025^*^
Gestational sac (mm)	-0.054	0.561
Crown rump length (mm)	-0.111	0.230

*r-Pearson Correlation Coefficient*

p-value>0.05 NS;

* p-value <0.05 S;

** p-value <0.001 HS

## DISCUSSION

This prospective cohort study was conducted in the Department of Obstetrics and Gynecology at the Ain Shams University Hospital (Special care unit of the fetus) from July 2019 to January 2020. A total of 120 healthy pregnant women attending our outpatient clinic at 6 to 12 weeks of gestation with no hormonal contraception or lactation in the last three cycles before pregnancy, aged 18-35 years, with a body mass index of 18-30 kg/m^2^ were included. Women with multiple pregnancies, uterine abnormalities, bleeding in early pregnancy, medical diseases with pregnancy (DM, HTN, etc.) and/or obstetric complications in previous pregnancies were excluded from our study. All healthy pregnant women with gestational ages between 6 and 12 weeks attending the special fetal care unit were interviewed (personal, present, past, menstrual and obstetric history) and underwent physical (general, abdominal and pelvic examinations) and ultrasound examination.

Sonography was performed using a Samsung H60 machine - convex Pro = CV1 = 8MHz, probe 4.9 MHz with patients with an empty bladder. A systematic approach was used. First, the uterus was scanned, then the adnexa, and finally the cul-de-sac. The gestational sac and yolk sac were identified. The inner yolk sac diameter was measured by placing calipers on inner margin. The yolk sac diameter was measured based on two cross sections in two dimensions. The range for a normal diameter was considered to be 2-5 mm. A large yolk sac was defined as a yolk sac with a diameter of more than 5mm, while a small yolk sac had a diameter of less than 2mm. The patients were followed up in routine antenatal care program until 16 weeks of gestation.

This prospective cohort study looked into the correlation between yolk sac diameter measured in transvaginal ultrasound examination at 6 to 12 weeks of gestation and adverse pregnancy outcomes. The patients were divided into three groups according to yolk sac diameter. Group 1 included yolk sac diameters <2mm (n=6); Group 2 included yolk sac diameters between 2-5mm (n=87); and Group 3 included yolk sac diameters >5mm (n=27). Statistical analysis found a highly statistically significant relationship between positive fetal life and negative fetal life according to yolk sac diameter, i.e., a high percentage of cases of positive fetal life occurred when a normal yolk sac diameter (2-5 mm) was present (*p*<0.001); in yolk sac diameters <2mm positive fetal life was 0.0% and negative fetal life was 42.9%; in yolk sac diameters of 2-5 mm positive fetal life was 81.1% and the negative fetal life was 7.1%; and in yolk sac diameters >5mm positive fetal life was 18.9% and negative fetal life was 50.0% (*p*<0.001), x^2^ 60.094; and the best cutoff value for yolk sac diameter was >0.56, with a sensitivity of 78.6%, a specificity of 84.3%, a positive predictive value of 74.7%, a negative predictive value of 87.1%, and a diagnostic accuracy of 78.2%.

The findings described by Tan *et al.* (2014) agreed with our results and established a significant relationship between yolk sac size and adverse pregnancy outcomes. The authors found that an enlarged yolk sac viewed before the 7^th^ week of gestation was strongly associated with a significantly increased risk of spontaneous miscarriage and presence of an echogenic or irregular yolk sac apparently unrelated to adverse perinatal outcomes. A total of 305 viable singleton pregnancies with gestational ages of 6 to 9 weeks were prospectively evaluated with respect to perinatal outcomes and yolk sac sonographic characteristics. In pregnancies with enlarged yolk sacs, miscarriages occurred in 37.5% of cases (3/8) (*p*=0.005) and the risk of miscarriage was statistically similar between pregnancies with regular and irregular yolk sacs (*p*=0.73).

Moradan & Forouzeshfar (2012) published results in line with ours and stated that abnormal yolk sac size was associated with spontaneous abortion. They considered the following yolk sac characteristics as normal: diameter: 2-5 mm; round shape; absence of degenerative changes; equal number with embryos; echogenic rim and hypoechoic center. Yolk sacs with diameters of less than 2mm or larger than 5mm were considered abnormal. A total of 191 cases were evaluated in patients divided into two groups; 22 (11.51%) cases had abnormal yolk sacs, and 14 of them (63.63%) ended in spontaneous abortions. In the control group, out of 169 (89.49%) cases, spontaneous abortion was seen in 6 (3.55%). There was a statistically significant difference in abortion rates between the two groups (*p*=0.000).

Cho *et al.* (2006) partially agreed with our results and stated that a very large yolk sac may exist in normal pregnancy. When embryonic heartbeats exist, the poor quality and early regression of a yolk sac are more specific than a large yolk sac in predicting pregnancy loss. When an embryo is undetectable, a relatively large yolk sac, even of normal shape, may be an indicator of miscarriage. Transvaginal ultrasonography was performed in 111 normal singleton pregnancies, 25 anembryonic gestations, and 18 missed abortions. In normal pregnancies with embryonic heartbeats, a deformed or absent yolk sac was never detected. Sequential appearance of the yolk sac, embryonic heartbeats and amniotic membrane were essential elements in normal pregnancy. The largest yolk sac in a viable pregnancy measured 8.1mm in diameter. Findings in anembryonic gestations included an absent yolk sac, an irregular-shaped yolk sac and a relatively large yolk sac (> 95% upper confidence limits, in 11 cases). In cases of missed abortion with prior existing embryonic heartbeats, abnormal findings included a relatively large, a progressively regressing, a relatively small, and a deformed yolk sac (an irregular-shaped yolk sac, an echogenic spot, or a band).

Rajani *et al.* (2012) agreed with our results and found that abnormal yolk sac size or shape and absence of a yolk sac may be used as indicators of poor outcome in early pregnancy, even before fetal morphology can be assessed in ultrasound examination. Ninety-five pregnant women selected randomly underwent TVS fortnightly from 5 to 11 weeks of gestation and at 12 weeks either TVUS or TAS was performed to identify changes in mean yolk sac diameter (MYSD) and yolk sac (YS) shape and pregnancy outcomes. In pregnancies with a normal outcome (n=72), MYSD grew gradually from 3.17mm at 5-5.2 weeks of GA to 5.03mm at 9-9.2 weeks of GA. At 11 weeks, the YS either disappeared (73.61%) or MYSD decreased (26.38%) with a yolk sac having a round and regular shape. A highly significant difference with a *p*-value <0.001 was detected between the MYSD in pregnancies with a normal outcome and missed abortions (n=19). Four cases of missed abortion had yolk sacs with an irregular shape. In anembryonic pregnancies, the YS was not seen. Srivastava *et al.* (2016) agreed with our study. The authors studied 72 pregnant women at 6-12 weeks of gestation. The mean yolk sac diameter was 3.7±1.8 mm. The smallest diameter of a yolk sac was 1.25mm and the largest was 8.96mm. Yolk sac size was normal in 62 (88.57%) cases and small in one (1.4%) case. In another seven (10%) cases, the yolk sac was abnormally enlarged. In cases where the yolk sac was either enlarged or smaller in size, gestation ended in miscarriage.

Rempen (1988) agreed with our results and stated that a yolk sac which is not visible in vaginal sonography between 5 and 10 complete weeks of menstrual age or a chorionic cavity diameter between 5 and 50mm and a yolk sac diameter above 6mm may serve as indicators of a developmental disturbance in early pregnancy. The detection rate and size of the yolk sac were evaluated with vaginal sonography in a prospective study including 377 singleton pregnancies. Normal pregnancies were seen in 298 cases and 79 ended in spontaneous abortions, the latter of which were viable in 18 cases at the time of examination and non-viable in 61 cases. With a reliable gestational age between 5 and 10 weeks, the yolk sac was recognized in 158 of 172 normal pregnancies (91.9%) and in all 14 viable later aborted pregnancies, but only in 10 of 29 non-viable pregnancies (34.5%) (*p*<0.000005). With a mean diameter of the chorionic cavity between 5 and 50 mm, the yolk sac was identified in 237 of 253 normal gestations (93.7%), in 16 of 18 viable but later aborted gestations (88.9%), but only in 14 of 41 non-viable gestations (34.1%) (*p*<0.0000001). A diameter of the yolk sac above 6mm was observed in 5 of 253 normal pregnancies 12.0%) and in 7 of 29 spontaneous abortions (24.1%) (*p*<0.0005). A diameter above 7mm was not seen in any instance of normal development and in four of pregnancies with pathological development (13.8%) (*p*<0.001).

Salamanca *et al.* (2013) published results in line with ours and established a correlation between morphology of conception and yolk sac appearance in cases of missed abortion. Two hundred consecutive cases of missed abortion/10 weeks diagnosed by transvaginal ultrasound were enrolled. In 104 gestations of embryos with morphological abnormalities, 88 (84.6 %) were at least one week smaller than expected for gestational age and 16 (15.4%) were of the expected size. From 32 normal morphologic embryos, seven (21.9%) were at least one week smaller than expected for gestational age, and 25 (78.1%) were of the expected size (*p*<0.005). Normal morphology embryos are linked more frequently with normal yolk sacs (62.5 %). Findings in anembryonic gestations (GD1) included an absent yolk sac (46.9%) and a cystic yolk sac (25%). Likewise, findings in GD2-3 embryos included more frequently a cystic yolk sac (42.9%) and an absent yolk sac (32.5%). GD4 embryos are associated with an echogenic yolk sac (40%), a relatively small hypoplastic YS (40%) and a relatively large cystic YS (20%). In DI embryos, the yolk sac was cystic (62.5%) or echogenic (37.5%).

Finally, Tan *et al*. (2011) disagreed with our results and stated that an irregular yolk sac shape and size were unrelated to increased risk of spontaneous abortion. The shape and size of the yolk sac were assessed by transvaginal sonography in 183 women who had normal healthy pregnancies with gestational ages of 6 to 8 weeks. Most of the embryos had a yolk sac with a regular shape (152 of 183 [83%]), whereas the remaining embryos had a yolk sac with an irregular shape (31 of 183 [17%]). Although there was a trend toward a lower rate of irregular yolk sacs with advancing gestational age, the difference was not statistically significant (*p*=0.13). Spontaneous abortion occurred in 6 of 183 pregnancies (3.3%): one of the 31 (3.2%) with an irregular yolk sac shape and five of the 152 (3.3%) with a regular yolk sac shape. Spontaneous abortion rates were statistically similar for pregnancies with a regular yolk sac shape and those with an irregular shape (*p*>.99).

We found a significant association between age and yolk sac size (ANOVA=9.265, *p*<0.001) and an insignificant association between yolk sac size and parity, BMI, blood pressure of first and second scan, gestational sac diameter in the first scan, crown rump length in the first scan, gestational age in the second scan, biparietal diameter in the second scan, abdominal circumference in the second scan, femur length in the second scan, and amniotic fluid index in the second scan. Tan *et al*. (2014) disagreed with our findings and described a moderately positive correlation between yolk sac size and gestational age, with yolk sac diameter increasing gradually with advancing gestational weeks (r=0.51, *p*<0.001). Rajani *et al.* (2012) disagreed with our results and found highly significant correlations (*p*-value <0.001) between MYSD and GA by LMP (r=+0.740), MYSD and MGSD (r=0.739), and MYSD and CRL (r=0.355) (*p* value <0.005).

### Strengths of the study

Every effort was made to ascertain that all data were correct, and only complete information was included in data analysis.

### Limitations of the study

To compare other measures of efficacy and safety would have required considerably more study participants; however, acquiring more subgroup data in a clinical trial setting may improve guidance on the use of TVUS measurement of yolk sac diameter for specific populations, the first scan performed between 6-12 weeks within a range of 6 weeks. Yolk sac size was measured by different sonographers. Patient follow-up was not performed at the same gestational age. We were unable to find a cutoff value for patients with a yolk sac <2mm because of their small number (n=6), which prevented it from attaining significance in the (ROC) curve.

## CONCLUSION

We found a highly significant correlation between yolk sac size and pregnancy outcome. Abnormalities of yolk sac size can be used as a good predictive indicator of early pregnancy loss, even before fetal morphology can be assessed in ultrasound examination.
